# Diabetes Ketoacidosis Complicating as Wrist Drop: A Case Report on Acute Motor Neuropathy

**DOI:** 10.7759/cureus.17081

**Published:** 2021-08-11

**Authors:** Shreya Arora, Shivani Gupta, Gautam Jesrani, Ruchika Saini, Monica Gupta

**Affiliations:** 1 General Medicine, Government Medical College and Hospital, Chandigarh, IND

**Keywords:** diabetic ketoacidosis, motor neuropathy, wrist drop, insulin, blood ketone

## Abstract

Diabetic ketoacidosis (DKA) is an acute and major complication of diabetes mellitus. Neurological complications can be seen at any time during the course of illness and range from decreased consciousness to ischemic or hemorrhagic stroke. Acute neuropathy is very rare in this milieu. Here, we report a case of a 40-year-old patient, who developed a left-sided wrist drop after being treated for DKA. The nerve conduction velocity studies demonstrated decreased action potential amplitude in only the motor component of the left radial nerve. Other possible causes of the complaint were ruled out and the patient was managed with cock-up splint, vitamin B1 and B6 supplementation, and physiotherapy. Despite all these measures, the patient had minimal improvement. Thus, close monitoring of patients is crucial to identify these infirmities, even after the acute condition has resolved.

## Introduction

Diabetic ketoacidosis (DKA) is a common and potentially life-threatening complication of diabetes mellitus, delineated by the triad of hyperglycemia, ketosis, and acidosis. It occurs due to a hormonal imbalance characterized by a relative or absolute insulin deficiency and an excess of insulin counter-regulatory hormones [1]. Amongst the various complications associated with this illness, neurological problems contribute most significantly to diabetes-related deaths. The most common neurological complication is cerebral edema, with an estimated risk of 6.8 per 1,000 cases of DKA [2]. Further, 10% of the total complications are due to ischemic and hemorrhagic strokes and the affliction is associated with significant mortality (24%) and morbidity (35%) [3]. Peripheral neuropathy as a consequence of DKA is extremely rare, and upper limb mono-neuropathy is even more so. In this report, we present a distinct case of acute upper limb mono-neuropathy presenting with wrist drop after being admitted and treated for DKA.

## Case presentation

A 40-year-old man was brought to the emergency department by his relatives with a history of altered consciousness for four hours. The patient was a known case of type-1 diabetes for the last 20 years and was taking injectable insulin to control his blood glucose. He had no other morbidity and there was no history of substance abuse. On examination, the patient had a deep respiratory pattern with 94% capillary oxygen saturation. His blood pressure was 106/68 mm Hg, blood glucose was 438 mg/dl, and Glasgow Coma Scale (GCS) score was 9/15 (eye-opening: 2/4, verbal response: 3/5, and motor response: 4/6). Blood ketone levels were measured immediately and found to be 4.1 mmol/L (normal <1.5). Normal saline at 10 ml/kg was instituted in the first hour as an initial measure for DKA and continued as per the guidelines. Arterial blood gas (ABG) analysis depicted a pH of 7.18, with an anion gap of 21 and potassium of 3.6 mEq/L, so the patient was subsequently shifted to the intensive care unit. A second intravenous line was secured for insulin infusion at 7 units/hour and his urine output was monitored alongside. Meanwhile, an electrocardiogram was done, which demonstrated sinus tachycardia of 112/min and chest x-ray showed no abnormality. With this management, his sensorium improved to 12/15 over the next three hours and he was completely conscious after 12 hours of in-patient management. After gaining consciousness, the patient admitted to being non-compliant with his insulin injections for the last two to three days.

On laboratory investigations, the patient had mild leukocytosis (13 × 10^9^/L, normal 4-11) with normal renal function tests, and urinalysis revealed no albuminuria or pus cells. Subsequent ABG measurements demonstrated improvement in the pH, but his potassium levels had decrement, therefore oral potassium was supplemented. The intravenous infusion was switched to subcutaneous insulin therapy on the second day as his pH fell into the normal range and the anion gap closed.

**Figure 1:**
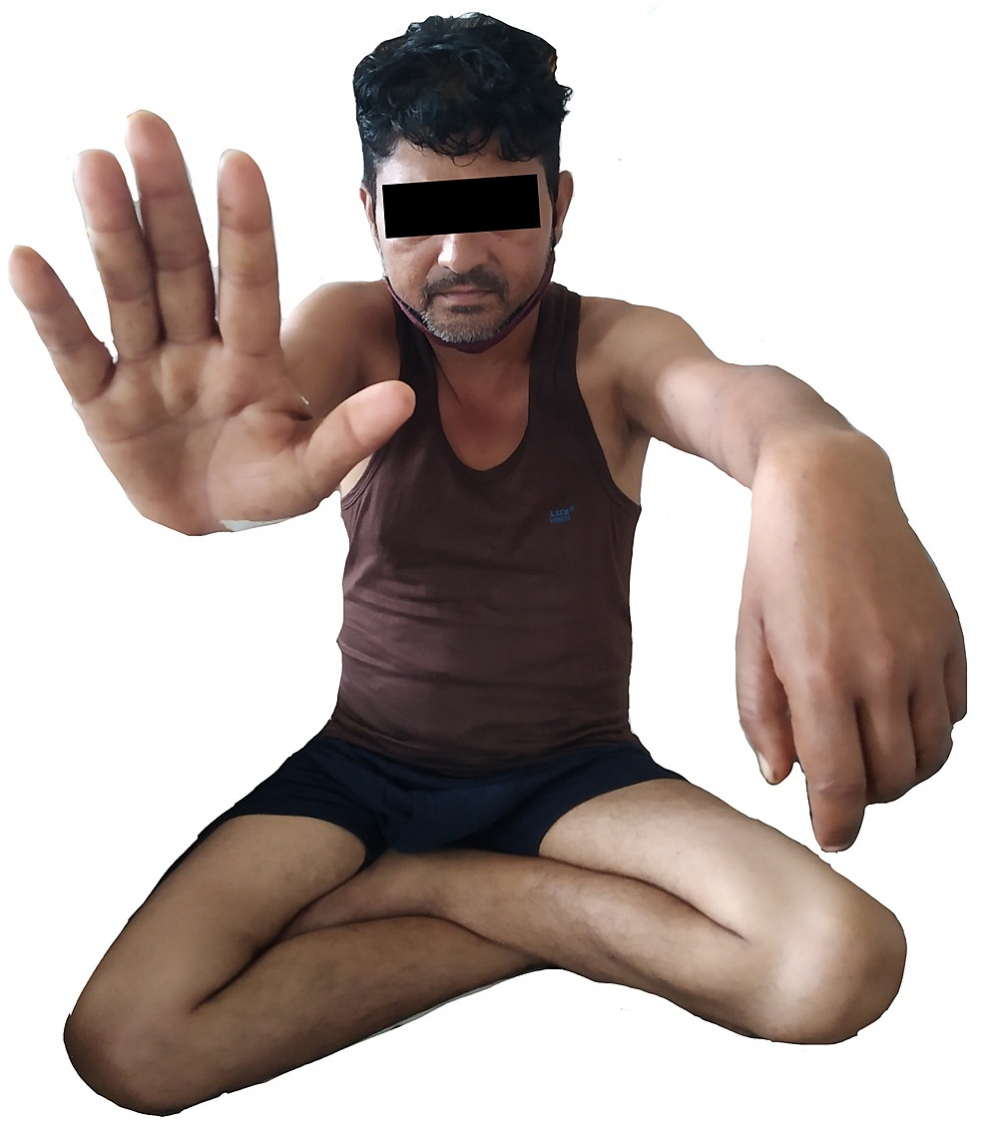
Hand drop on the left side with normal movement on the right side.

**Figure 2:**
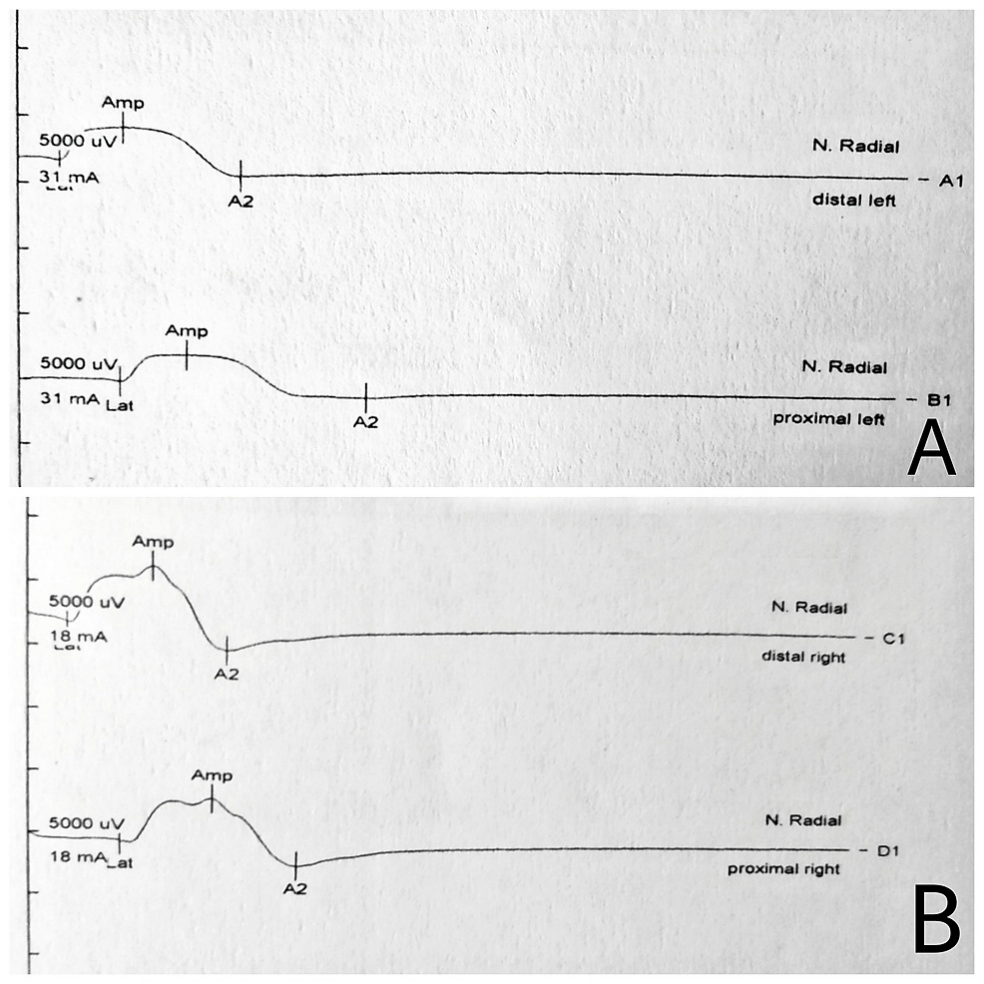
Radial nerve motor action potential recording demonstrating decreased amplitude on the left side (A), compared with the right side (B).

On the third day of admission, the patient started experiencing weakness in his left hand and was unable to hold things with appropriate grip. He had no history of recent trauma, abnormal sleeping positions, intermittent abdominal pain, or previous limb weakness. Local sensory symptoms, pain, tenderness, or signs of compartment syndrome were absent around the left wrist. There was no restriction on passive movements of the elbow, wrist, or fingers, but active movements were reduced (Figure 1). The diagnosis of wrist drop was suspected. The right upper limb and both the lower limbs showed no alterations and his cranial nerve examination was normal. His electrolytes including potassium, calcium, and magnesium levels were re-checked but found to be within the normal range. Further, brain and cervical magnetic resonance imaging showed no abnormalities. Nerve conduction study demonstrated left-sided radial isolated motor neuropathy with decreased action potential amplitude (Figure 2). His blood film had no basophilic stippling, and vitamin B12, folic acid, and thyroid function tests were normal. Management was comprised of cock-up splint plus vitamin B1 and B6 supplementation. Despite this, no improvement was seen in wrist movements, but his finger extension improved minimally. He was subsequently discharged with the advice of physiotherapy rehabilitation. At two months follow-up, no wrist improvement was achieved, for which a permanent disability was suspected.

## Discussion

Diabetic neuropathy (DN) is a common disorder and is defined by the presence of signs and symptoms of peripheral nerve dysfunction in a patient with diabetes mellitus in whom all other causes of peripheral nerve dysfunction like vitamin deficiency, infection, inflammation, toxins, autoimmune, paraneoplastic, and genetic have been excluded [4]. Although the prevalence of DN in India is not known for certain, a recent study conducted in South India estimated it to be 39.3% [5]. Various neuropathy syndromes may be encountered in patients with diabetes (Table 1) [6]. According to a study by Yagihashi et al., an increased flux of the polyol pathway regulated by aldose reductase activation, enhanced advanced glycation end-products formation, excessive release of cytokines, activation of protein kinase C, and exaggerated oxidative stress are some of the components of the metabolic cascade initiated by prolonged hyperglycemia that ultimately lead to peripheral nerve ischemia [7].

**Table 1:** Classification of the diabetic neuropathies.

Symmetric involvement
Autoimmune autonomic ganglionopathy or neuropathy (gradual onset)
Painful sensory neuropathy (gradual onset)
Diabetic polyneuropathy (gradual onset)
Insulin neuritis (acute onset)
Hypoglycemia induced neuropathy (acute onset)
Polyneuropathy after ketoacidosis (acute onset)
Asymmetric involvement
Cranial neuropathies (acute onset)
Limb neuropathies (acute onset): median, ulnar, peroneal, obturator, and lateral cutaneous nerve of the thigh
Radiculo-plexus neuropathies (subacute onset): cervical, brachial, and lumbosacral

Distal symmetrical polyneuropathy is the commonest type of DN and possibly accounts for 75% of all cases [8]. It can involve either the large or small fibers, or both, and affect the sensory or motor system. Sensory impairment usually occurs in glove and stocking distribution. It develops over years in patients with long-standing hyperglycemia. Mono-neuropathy in DM may have a sudden onset and most frequently involves the median (5.8% of all DN), ulnar (2.1%), radial (0.6%), and common peroneal nerves (0.1%) [9]. Our patient developed acute motor mono-neuropathy of the left radial nerve manifesting as wrist drop as a complication of DKA, suggested by the temporality and exclusion of all other causes. There are limited reports of peripheral nervous system involvement during periods of DKA or on the pathogenesis of mono-neuropathies following DKA. It is hypothesized to develop as a result of peripheral nerve ischemia, transient pro-coagulant state, or other hemodynamic and metabolic abnormalities occurring during the course of this acute illness [10]. Moreover, the diagnosis of this entity is primarily clinical, supported by nerve conduction studies and exclusion of other potential causes as followed in this report.

Treatment of DN includes tight glycemic control and modification of associated cardiovascular risk factors, including raised serum triglyceride, body mass index, smoking, and hypertension [11]. Based on the plausible mechanisms of neuropathy, treatment options also include aldose reductase inhibitors (tolrestat and zenarestat), strong antioxidants such as alpha-lipoic acid, and benfotiamine, a derivative of vitamin B1, which has been shown to increase the utilization of active glycolysis products [12-14].

## Conclusions

Although cerebral manifestations are common neurological complications of DKA and are even a major cause of mortality, peripheral neuropathy is one of the paramount causes of disability in diabetic patients. This report illustrates a case of DKA who subsequently complicated with acute radial motor neuropathy after the management of his principal malady. Despite the timely diagnosis and adequate treatment, there was minor improvement in his hand debility and the patient was left with a permanent impairment. This emphasizes the need to monitor patients with DKA for the development of neurological complications, even after their blood glucose levels have normalized.
